# Relationship between subclasses low-density lipoprotein and carotid plaque

**DOI:** 10.1515/tnsci-2022-0210

**Published:** 2022-03-03

**Authors:** Zhanhai Pan, Huiwen Guo, Qingqing Wang, Sha Tian, Xiaoxuan Zhang, Chengbo Li, Zheng Ma

**Affiliations:** Department of Neurology, Affiliated Hospital of Chengde Medical College, Chengde City, Hebei Province, China

**Keywords:** unstable plaque, low-density lipoprotein subclasses, small-dense low-density lipoprotein

## Abstract

**Backgound:**

Low-density lipoprotein (LDL) cholesterol can lead to the occurrence of atherosclerotic plaques, but patients with normal LDL still have atherosclerotic plaques in clinical practice. With the proposal of LDL subclass, this experiment investigated the relationship between the LDL content of different subclasses and the stability of carotid plaques.

**Methods:**

Plaque stability was suggested by carotid ultrasound results. 37 patients with stable plaques were classified into one group and 41 patients with unstable plaques were classified into another group. The data of age, glycosylated hemoglobin (Ghb), and homocysteine (Hcy) were collected. The contents of LDL subclasses were measured by LIPOPRINT system. The data of total cholesterol (TC), LDL cholesterol, and triglyceride (TG) were collected. The plaque stability was assessed by carotid artery color Doppler ultrasound and the intima-media thickness (IMT) was measured.

**Results:**

The levels of LDL-1 subclass 19.00 (13.00, 27.50) and LDL-2 subclass (21.62 ± 7.24) in the stable plaque group were higher than those in the unstable plaque group (*p* < 0.05). The levels of LDL-3 subclass (12.24 ± 4.58), LDL-4 subclass 5.00 (2.00, 9.00), and sd-LDL 0 (0.00, 3.00) in the unstable plaque group were higher than those in the stable plaque group (*p* < 0.05). LDL-1 subclass (adjusted OR = 0.923 and *p* < 0.05), and LDL-3 subclass (adjusted OR = 1.176 and *p* < 0.05) were independent risk factors for plaque stability.

**Conclusion:**

Elevated LDL1 is associated with stable plaques whereas LDL3 was found associated with unstable plaques.

## Abbreviation

AS: Atherosclerosis
AIS: cute ischemic stroke
BMI: body mass index
Ghb: glycosylated hemoglobin
Hcy: homocysteine
IMT: intima-media thickness
LDL: low-density lipoprotein
LPS: lipopolysaccharide
TC: total cholesterol
TG: triglyceride

## 1 Introduction

Atherosclerosis (AS) is a pathological change characterized by the formation of porridge tumors or fibrous plaques in the vascular intima and the continuous progression of systemic arterial vessels. With the continuous progression of lesions, pathological processes such as grease pattern, fibrous plaques, and atheromatous plaques may occur, and fat streaks on the arterial wall gradually develop into atherosclerotic plaques. Atherosclerotic plaques can be divided into stable plaques and unstable plaques according to the characteristics of size of the lipid pool, thickness of fibrous cap, and the presence of inflammation in plaques [1]. The vulnerable plaques are characterized by thin fibrous cap, large necrotic core, and plaque inflammation. The carotid artery is one of the vessels most likely to develop AS [2]. It is currently believed that carotid atherosclerotic plaque rupture, embolus shedding, and *in situ* thrombosis that in turn cause severe stenosis or embolism of distal vessels are the main pathogenesis of acute ischemic stroke (AIS). Unstable carotid atherosclerotic plaques are more likely to lead to AIS than stable plaques [3]. There are many common risk factors for carotid AS, such as plasma low-density lipoprotein (LDL) cholesterol, homocysteine (Hcy), hypertension, and diabetes. In addition, recent studies have shown that gut bacteria, immunopathogenesis also affect atherosclerosis[4]. Many domestic and foreign studies have shown that LDL is one of the most important risk factors for AS [5]. However, in daily clinical practice, we find that many patients with carotid atherosclerotic plaques suggested by carotid artery color Doppler ultrasound have plasma LDL cholesterol levels within or lower than the normal range. The proposal of LDL subclasses provides a hypothetical theoretical basis for this phenomenon. LDL cholesterol in blood lipids has obvious heterogeneity in density and diameter. According to the heterogeneity of LDL cholesterol, LDL can be classified into 1–7 different subclasses [6]. Different subclasses of LDL have different effects on the occurrence and development of carotid AS. Relevant studies have shown that 3–7 subclasses of LDL can be classified into one class and named small-dense low-density lipoprotein (sd-LDL) due to their similar particle diameter size and oxidation [7]. Moreover, sd-LDL has a promoting effect on the formation of atherosclerotic plaques. However, there is no report on the correlation between different LDL subclasses and the stability of carotid atherosclerotic plaques. Hence, this study examined this correlation.

## 2 Materials and methods

### 2.1 Patients

The patients who were hospitalized in the Department of Neurology, Affiliated Medical College of Chengde Medical College between July 2019 and August 2020 were included. The patients with stable plaques confirmed by carotid ultrasound were included into the stable plaque group (*n* = 37), comprising 24 males and 13 females, with an average age of 64.84 ± 10.10 years. The patients with unstable plaques confirmed by carotid ultrasound were included into the unstable plaque group (*n* = 41), comprising 25 males and 16 females, with an average age of 65.61 ± 9.53 years. All patients met the inclusion criteria.


**Informed consent:** Informed consent has been obtained from all individuals included in this study.
**Ethical approval:** The research related to human use has been complied with all the relevant national regulations, institutional policies, and in accordance with the tenets of the Helsinki Declaration, and has been approved by the authors’ institutional review board or equivalent committee.

### 2.2 Inclusion and exclusion criteria

Inclusion criteria: (1) patients who could cooperate to improve carotid ultrasound, and ultrasound results suggested the presence of carotid atheromatous plaque; (2) blood lipid examination showed LDL cholesterol ≤3.36 mmol/L; (3) the diagnosis of acute cerebral infarction based on the “Chinese Guidelines for the Diagnosis and Treatment of Acute Ischemic Stroke 2018” diagnostic criteria for acute cerebrovascular disease, auxiliary examination excluded acute cerebral infarction.

Exclusion criteria: (1) blood lipid examination showed LDL cholesterol >3.36 mmol/L, or the patient took lipid-regulating drugs within 12 months before blood test; (2) concomitant cerebral infarction, cerebral hemorrhage, and other acute cerebrovascular disease; (3) concomitant atrial fibrillation, acute coronary syndrome, or connective tissue and other immune diseases; (4) severe liver and kidney dysfunction.

### 2.3 Observed indicators

(1) The basic data of patients including age, gender, history of hypertension, history of smoking, history of drinking, glycosylated hemoglobin (Ghb), hcy, and body mass index (BMI) were recorded.
(2) Blood lipids were tested. After the patients fasted for >12 h, 4 mL of venous blood was collected and placed in EDTA anticoagulant tubes. The blood samples were slowly mixed, centrifuged at 3,000 rpm for 5 min, and 1.5 mL of plasma was taken. CardioChek PA cholesterol detector was used to detect the levels of total cholesterol (TC), triglyceride (TG), and LDL cholesterol in the plasma, and the detection reagent was purchased from Shanghai Mapui Biotechnology Co., Ltd. Next 50 µL of plasma and 10 µL of staining solution were vortexed for 30 s to ensure thorough mixing, and the mixture was placed in 35°C water bath for 15 min. The gel electrophoresis was performed with the mixture using DYY-10C electrophoresis apparatus power supply purchased from Beijing Liuyi Biotechnology Co., Ltd and DYCZ-27B electrophoresis apparatus. The above test results were controlled using the original quality control kit of the manufacturing company.
(3) Carotid plaque stability and thickness measurement: Carotid ultrasound examination was performed using Sono M-Turbo and Sono Edge model color Doppler ultrasound machines. 5–10 MHz linear array probes were used for normal patients, and 2–5 MHz convex array probes or 5–8 MHz small convex array probes were used for some obese patients with high carotid bifurcation position, deep vascular position, and body size. The subject was supine on the examination bed, and both carotid arteries were transected and explored longitudinally with a probe. The common carotid artery, carotid bifurcation, and internal carotid artery were explored and the pictures were saved, respectively. Measurement of carotid plaque thickness: Plaque thickness was expressed by intima-media thickness (IMT). When the measured IMT ≥ 1.2 mm, carotid atherosclerotic plaque formation was considered [8]. The presence of plaques in the common carotid artery, carotid bulb, and internal carotid artery was observed. If there were multiple plaques, the mean plaque thickness was calculated. The plaque thickness was recorded. Plaque stability assessment: Plaque ultrasound echo characteristics reflect plaque stability, and isoechoic plaques are considered to contain certain solid components, such as fibrous tissue. Heteroechoic plaques usually contain histological features of plaque instability, such as hemorrhages and intraplaque hemorrhages [9]. Heteroechoic plaques are considered to have a higher risk of rupture and thrombosis. Therefore, in this study, isoechoic plaques were classified as stable plaques and heterogeneous echogenic plaques as unstable plaques. The patient’s carotid plaque stability results were included. The differences in outcome measures between patients with different stable plaques were compared.

### 2.4 Statistical analysis

SPSS 26.0 statistical software was used for data processing and analysis. The variable data were tested linearly using the *W*-test method. Normally distributed data were represented as *X* ± *s* and analyzed with two independent samples *t*-test. Non-normally distributed data were expressed as median (first quartile and third quartile) [M (P25 and P75)], and analyzed using Mann–Whitney *U* test. Spearman’s test was used to analyze the correlation of the data; binary logistic regression was used to analyze the data; and ROC curve was applied to analyze the sensitivity of the results to the examined factors.

## 3 Results

### 3.1 Comparison of basic data

There were no statistically significant differences in gender, history of hypertension, history of smoking, history of drinking, age, TC, LDL cholesterol, TG, Ghb, Hcy, BMI, and mean IMT between the two groups (*p* > 0.05) (Tables 1 and 2).

**Table 1:** Comparison of gender, smoking, alcohol, and hypertension

Project	Gender (*n*)		Smoking (*n*)		Alcohol (*n*)		Hypertension (*n*)	
	Male	Female	Yes	No	Yes	No	Yes	No
Vulnerable plaque	24	13	16	21	8	29	24	13
Stable plaque	25	16	18	23	12	29	27	14
*χ* ^2^	0.126		0.003		0.596			0.008
*p*	0.723		0.953		0.440			0.927

Note: There were no significant differences in constituent ratio of gender, smoking, alcohol, and hypertension between the two groups with different arterial plaque stability (*p* > 0.05).

**Table 2:** Comparison of basic data

Project	Vulnerable plaque	Stable plaque	*T*/*Z*	*p*-value
	(*n* = 41)	(*n* = 37)		
Age (year)	65.61 ± 9.53	64.84 ± 10.10	0.347	0.729
TC (mmol/L)	4.05 ± 0.71	4.03 ± 0.66	0.153	0.878
LDL (mmol/L)	2.10 ± 0.46	2.31 ± 0.55	−1.837	0.07
TG (mmol/L)	1.21 (0.97, 1.67)	1.36 (1.02, 1.74)	−0.831	0.406
Ghb (%)	5.80 (5.60, 6.60)	5.80 (5.40, 6.95)	−0.536	0.592
Hcy (mmol/L)	13.00 (11.40, 15.20)	13.90 (11.00, 18.65)	−0.506	0.613
IMT (cm)	0.21 (0.19, 0.24)	0.20 (0.17, 0.24)	−1.2605	0.207

Note: There were no significant differences in baseline data between the two groups with different arterial plaque stability (*p* > 0.05).

### 3.2 Comparison of levels of different LDL subclasses between the two groups

**Figure 1:**
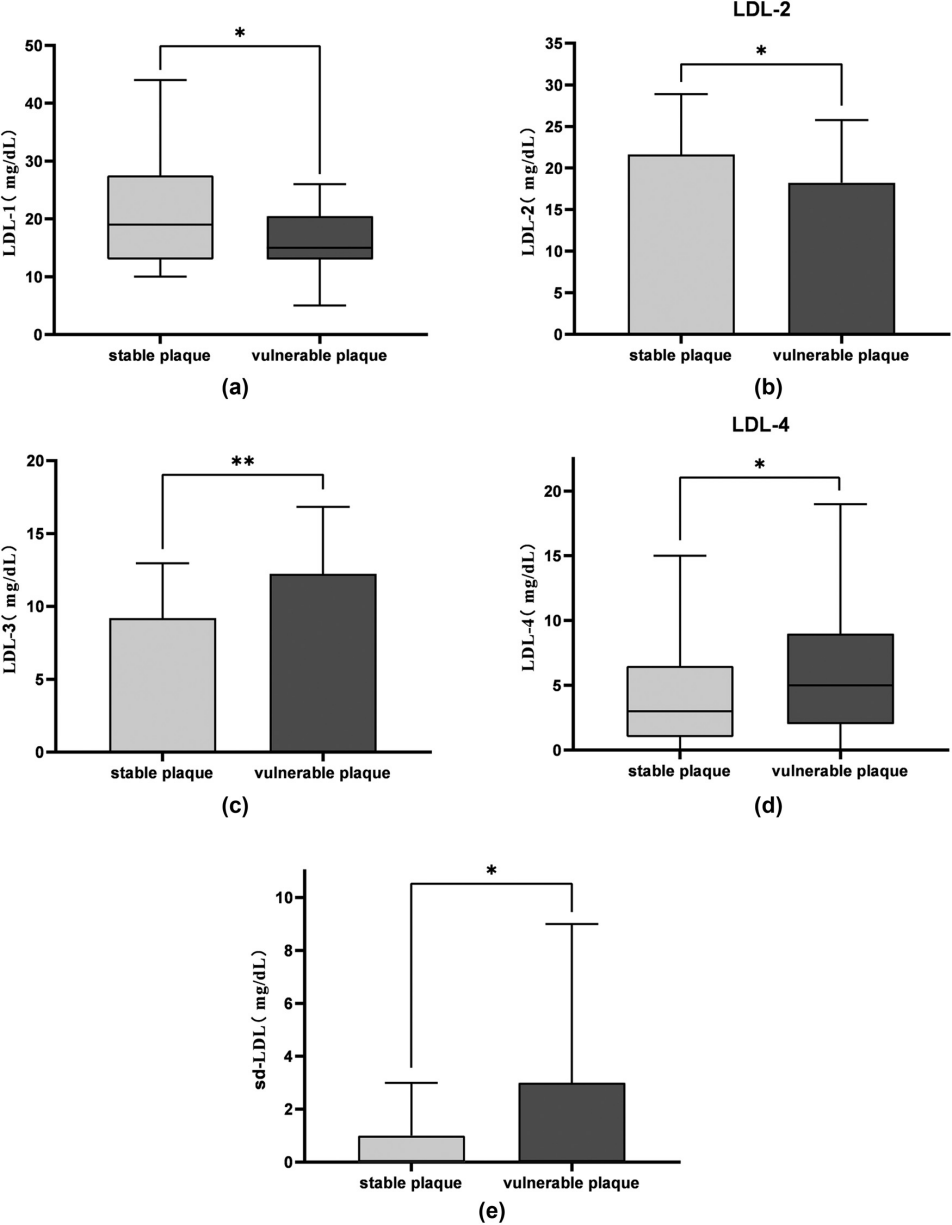
Differences in LDL subclasses between the two groups. * *p* < 0.05 and ** *p* < 0.01. (a and b) LDL-1 and LDL-2 were more abundant in the stable plaque group. (c–e) LDL-3, LDL-4, and sd-LDL were more abundant in the unstable plaque group.

Recent studies have shown that LDL-3–7 subclasses are classified as sd-LDL due to their similar lipoprotein particle size [10]. In this study, LDL-3 subclass and LDL-4 subclass were separately analyzed. Combining the results of blood lipid tests, it was found that the levels of LDL-5–7 subclasses were very low, and the test results of some patients suggested that they did not contain the above LDL subclasses. Therefore, LDL-5–7 subclasses were classified as sd-LDL in this study. There were significant differences in the levels of each subclass of LDL in patients with different plaque stability (*p* < 0.05). The levels of LDL-1 and LDL-2 in patients with stable plaques were higher than those in patients with unstable plaques, and the levels of LDL-3, LDL-4, and sd-LDL in patients with unstable plaques were higher than those in patients with stable plaques (Table 3 and Figure 1).

**Table 3:** Comparison of levels of different LDL subclasses between the two groups

	Vulnerable plaque (*n* = 41)	Stable plaque (*n* = 37)	*T*/*Z*	*p*-value
LDL-1 (mg/dL)	15.00 (13.00, 20.50)	19.00 (13.00, 27.50)	−2.226	0.026*
LDL-2 (mg/dL)	18.22 ± 7.54	21.62 ± 7.24	−2.028	0.046*
LDL-3 (mg/dL)	12.24 ± 4.58	9.19 ± 3.77	3.195	0.002*
LDL-4 (mg/dL)	5.00 (2.00, 9.00)	3.00 (1.00, 6.50)	−2.02	0.043*
sd-LDL (mg/dL)	0.00 (0.00, 3.00)	0.00 (0.00, 1.00)	−2.342	0.019*

*p < 0.05. Note: Levels of different LDL subclasses were significantly different between the two groups (*p* < 0.05). LDL-1 and LDL-2 were more abundant in the stable plaque group; LDL-3, LDL-4, and sd-LDL were more abundant in the unstable plaque group.

### 3.3 Effects of different LDL subclasses on plaque stability

**Figure 2:**
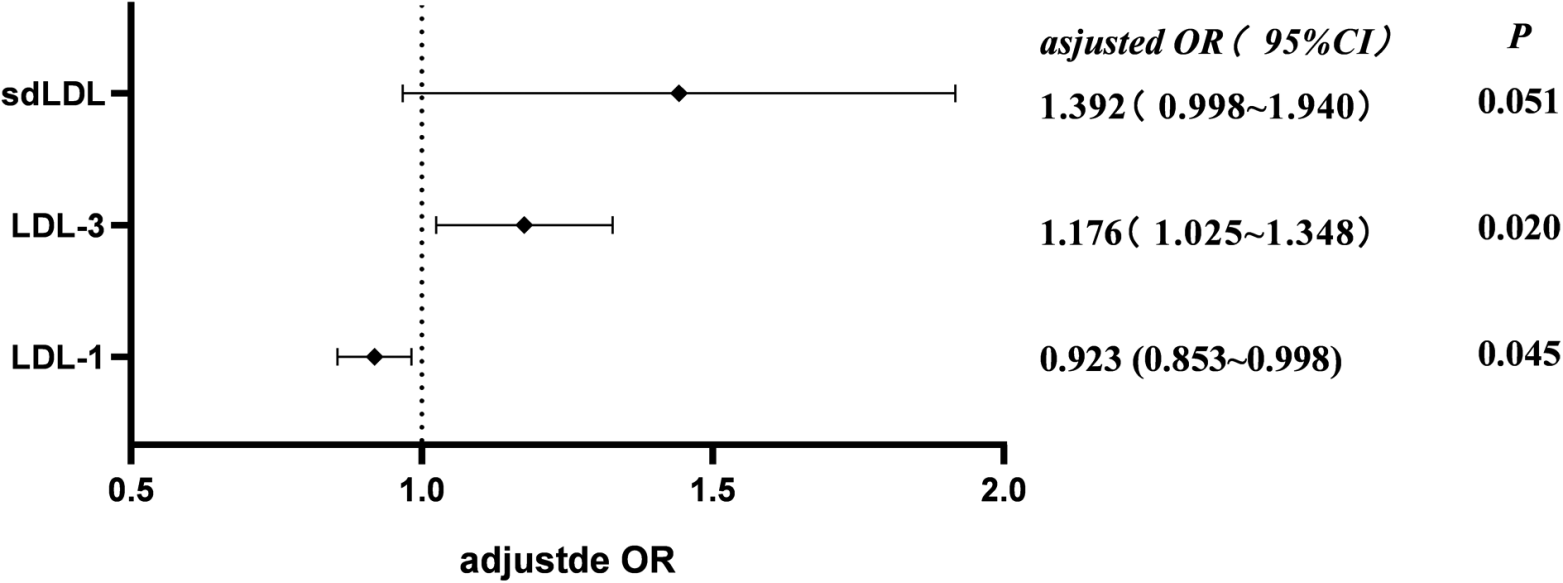
Adjusted OR for risk factors of plaque stability.

Univariate screening of the indicators to be tested revealed a correlation between the levels of LDL-1 (rs = −0.338 and *p* < 0.01), LDL-2 (rs = −0.227 and, *p* < 0.05), LDL-3 (rs = 0.344 and *p* < 0.01), and sd-LDL (rs = 0.320 and *p* < 0.01) and plaque stability. Binary logistic regression showed that LDL-1 (crude OR = 0.905 and *p* < 0.01), LDL-3 (crude OR = 1.202 and *p* < 0.01), and sd-LDL (crude OR = 1.560 and *p* < 0.05) had an effect on plaque stability, but the degree of effect of sd-LDL (adjusted OR 95% CI [0.998–1.940] and *p* > 0.05) on plaque stability was not statistically significant (*p* > 0.05), while LDL-1 (adjusted OR = 0.923 and *p* < 0.05) and LDL-3 (adjusted OR = 1.176 and *p* < 0.05) were independent risk factors for plaque stability (Table 4 and Figure 2).

**Table 4:** Effect of different LDL subclasses on plaque stability

	LDL-1	LDL-2	LDL-3	Sd-LDL
Crude P	0.006*	0.051	0.005*	0.011*
Crude B	−0.100	−0.064	0.184	0.444
Crude OR	0.905	0.938	1.202	1.560
(95% CI)	(0.842–0.972)	(0.880–1.000)	(1.058–1.367)	(1.107–2.198)
Adjusted P	0.045*		0.020*	0.051
Adjusted B	−0.081		0.162	0.330
Adjusted OR	0.923		1.176	1.392
(95% CI)	(0.853–0.998)		(1.025–1.348)	(0.998–1.940)

*p < 0.05. Note: After exclusion of confounding factors, the adjusted OR for sd-LDL was significant at *p* > 0.05, but was not an independent risk factor for plaque stability. The adjusted OR for LDL-1 and LDL-3 were significant at *p* < 0.05, and were independent risk factors for plaque stability.

### 3.4 Accuracy of LDL-1 and LDL-3 for diagnosis of plaque stability

The accuracy of LDL-1 for diagnosis of stable plaques was 64.6% (AUC = 0.646 and *p* < 0.05). Plaques tended to stabilize when the LDL-1 level was >21.5 mg/dL. The accuracy of LDL-3 for diagnosis of unstable plaques was 69.6% (AUC = 0.696 and *p* < 0.01). The plaque was more likely to be unstable when the level of LDL-3 was >10.5 mg/dL (Table 5).

**Table 5:** Accuracy of LDL-1 and LDL-3 for diagnosis of plaque stability

	AUC	95% CI	*p*-value	Se%	Sp%	Cut-off point
LDL-1 (mg/dL)	0.646	0.522–0.771	0.026*	43.24	85.37	21.50
LDL-3 (mg/dL)	0.696	0.580–0.813	0.003*	70.73	59.46	10.50

*p < 0.05. Note: The accuracy of LDL-1 in the diagnosis of stable plaques was 64.6%, and plaques tended to stabilize when the LDL-1 level was >21.5 mg/dL. The accuracy of LDL-3 in the diagnosis of unstable plaques was 69.6%, and the plaques tended to be unstable when the LDL-3 level was >10.5 mg/dL.

## 4 Discussion

Cardiovascular and cerebrovascular diseases seriously endanger the health of the middle-aged and elderly populations worldwide. Global survey results showed that AIS was highest in China, seriously endangering the physical and mental health of the Chinese [11]. Large artery AS is the main cause of AIS. Landray and other scholars have shown that isolated carotid artery disease is more prevalent in Asia [12], which in turn leads to the occurrence of ischemic stroke.

There are many risk factors for AIS, and the degree of carotid artery stenosis is a clear risk factor for stroke. Studies have shown that LDL cholesterol promotes the formation and progression of atherosclerotic plaques, resulting in the formation of vascular stenosis [13]. LDL refers to lipoproteins with a density of 1.019–1.063 g/mL, and each lipoprotein contains one apolipoprotein B molecule. In this study, the LIPOPRINT lipoprotein classification detection system was applied to subdivide LDL into seven different types. Studies have shown that different LDL subclasses have different effects on plaques. Hence, compared with the total content of LDL, different LDL subclasses have better risk assessment for atherosclerotic plaque formation and are more accurate biomarkers [14,15]. In addition, the occurrence of unstable plaques is more likely to have adverse events such as rupture, bleeding, and thrombosis, which is one of the important factors leading to ischemic cerebrovascular disease [16]. Therefore, plaque stability can predict the occurrence of stroke, independent of the degree of stenosis [1]. The pathological characteristics affecting plaque stability include fibrous cap state, erosion and ulcer, core state, intraplate hemorrhage or neovascularization, intraplate hemorrhage, and the degree of plaque inflammation [8,17]. Similarly, exploring the effect of different LDL subclasses on plaque stability is more conducive for the prevention of stroke.

Michal et al. showed a correlation between LDL levels and plaque stability [18]. According to the heterogeneity of LDL cholesterol particles, they can be divided into seven different types. According to the characteristics of lipoprotein diameter, density, and oxidation, they can be divided into two categories: LDL-1 and LDL-2 particles are large, called large-buoyant low-density lipoprotein (lb-LDL), and LDL-3–7 particles are small, called sd-LDL [19]. Studies have shown that sd-LDL particles are more likely to lead to AS than lb-LDL particles [7]. However, the stability of each subclass and plaque needs to be further explored.

This study found that different LDL subclasses have different effects on plaque stability. Among them, the LDL-3–7 subclasses were more likely to undergo various atherogenic modifications, such as deacetylation, glycation, and oxidation, in the blood circulation compared with LDL cholesterol, which further increases its atherogenic capacity. The decreased sialic acid content of sd-LDL leads to its significant deacetylation, which in turn increases its adhesion ability to the vascular wall [20]. Moreover, sd-LDL cholesterol particles have a smaller diameter and hence are more likely to penetrate vascular endothelial cells, promote the infiltration and accumulation of endothelial cells, and form a larger core; the sd-LDL sub-fraction contains fewer components of β-carotene, which has antioxidant effect, so this component is more likely to form oxidized lipoproteins [21], mediate the activation of macrophages to promote the production of pro-inflammatory cytokines, oxygen free radicals and foam cells, and exacerbate the degree of inflammatory response; the low affinity of sd-LDL for LDL receptors in the liver leads to a decrease in the clearance rate of LDL particles, a longer half-life, and a more persistent damage to vascular endothelial cells [22]. The above mechanism affects the physiological characteristics of plaques, such as the core of plaques, the degree of plaque inflammation, and the status of fibrous cap, and reduces the stability of plaques. In addition, gut bacteria also have an effect on AS, and gut bacteria promote the process of AS by forming lipopolysaccharide (LPS) [4]. LPS enhances the uptake of ox-LDL by macrophages by enhancing the expression of scavenger receptors, and LPS and ox-LDL act together to promote the conversion of macrophages into typical foam cells, which in turn exacerbates atherosclerosis [23]. Sd-LDL is more easily oxidized to form ox-LDL, promoting the above process to form unstable plaques. The results of the study also confirmed this inference, and the LDL-3 in the sd-LDL fraction was an independent risk factor for plaque stability and is able to promote the formation of unstable plaques. However, the specific effect of lb-LDL on arterial plaque formation remains controversial. Zitnanova et al. showed that LDL-2 had a positive correlation with antioxidant enzymes, so it had a protective effect on AS [14]. However, another study found that LDL-2 could promote AS [15], and LDL-2 was correlated with plaque stability, without causal relationship. Therefore, the effect of this subclass on arteriosclerosis remains controversial. In terms of the effect of LDL-1 on plaque stability, Zitnanova et al. showed that LDL-1 was positively correlated with the activity of antioxidant enzymes, so it had the characteristics of anti-AS. Combined with the characteristics of large diameter and weak oxidation of LDL-1 particles [19], it can be deduced that LDL-1 can inhibit the formation of unstable plaques, and this inference is consistent with the results of this study. Combined with the results of this study, it can be concluded that LDL-1 is an independent risk factor for plaque stability and is able to promote the formation of stable plaques. The stability of other LDLs to plaque needs further study.

## 5 Conclusion

Different LDL subclasses have different effects on plaque stability because of different structures and biological characteristics. This study showed that the independent risk factors of plaque stability only included LDL-1 and LDL-3. The LDL-1 promotes the formation of stable plaques and the LDL-3 promotes the formation of unstable plaques, so more LDL-1 and less LDL-3 help to prevent the occurrence and development of unstable atherosclerotic plaques.
